# Impact of Early versus Late Initiation of Renal Replacement Therapy in Patients with Cardiac Surgery-Associated Acute Kidney Injury: Meta-Analysis with Trial Sequential Analysis of Randomized Controlled Trials

**DOI:** 10.1155/2018/6942829

**Published:** 2018-12-18

**Authors:** Jie Cui, Da Tang, Zhen Chen, Genglong Liu

**Affiliations:** ^1^Head and Neck Surgery, Affiliated Cancer Hospital & Institute of Guangzhou Medical University, 510095 Guangdong Province, China; ^2^Department of General Surgery, Third Xiangya Hospital, Central South University, Changsha 410008, Hunan Province, China; ^3^Intensive Care Unit, Shunde Hospital, Southern Medical University, Foshan 528300, Guangdong Province, China; ^4^Department of Pathology, Affiliated Cancer Hospital & Institute of Guangzhou Medical University, Guangzhou 510095, Guangdong Province, China

## Abstract

**Background:**

Previous studies have examined the effect of the initiation time of renal replacement therapy (RRT) in patients with cardiac surgery-associated acute kidney injury (CSA-AKI), but the findings remain controversial. The aim of this meta-analysis was to systematically and quantitatively compare the impact of early versus late initiation of RRT on the outcome of patients with CSA-AKI.

**Methods:**

Four databases (PubMed, the Cochrane Library, ISI Web of Knowledge, and Embase) were systematically searched from inception to June 2018 for randomized clinical trials (RCTs). Two investigators independently performed the literature search, study selection, data extraction, and quality evaluation. Meta-analysis and trial sequential analysis (TSA) were used to examine the impact of RRT initiation time on all-cause mortality (primary outcome). The Grading of Recommendations Assessment Development and Evaluation (GRADE) was used to evaluate the level of evidence.

**Results:**

We identified 4 RCTs with 355 patients that were eligible for inclusion. Pooled analyses indicated no difference in mortality for patients receiving early and late initiation of RRT (relative risk [RR] = 0.61, 95% confidence interval [CI] = 0.33 to 1.12). However, the results were not confirmed by TSA. Similarly, early RRT did not reduce the length of stay (LOS) in the intensive care unit (ICU) (mean difference [MD] = -1.04; 95% CI = -3.34 to 1.27) or the LOS in the hospital (MD = -1.57; 95% CI = -4.62 to 1.48). Analysis using GRADE indicated the certainty of the body of evidence was very low for a benefit from early initiation of RRT.

**Conclusion:**

Early initiation of RRT had no beneficial impacts on outcomes in patients with CSA-AKI. Future larger and more adequately powered prospective RCTs are needed to verify the benefit of reduced mortality associated with early initiation of RRT.

**Trial Registration:**

This trial is registered with PROSPERO registration number CRD42018084465, registered on 11 February 2018.

## 1. Background

Cardiac surgery-associated acute kidney injury (CSA-AKI) is the most common clinically severe complication in adult patients following cardiac surgery [1]. In particular, acute kidney injury (AKI) occurs in 5% to 42% of patients after cardiac surgery (depending on how AKI is defined) and is associated with increased morbidity, length of hospital stay, cost of care, and mortality [2–5]. Considering the poor prognosis of these patients and the significant costs for their medical care, urgent treatment is necessary. Renal replacement therapy (RRT) is the main life-saving treatment for these patients [6, 7], because it prolongs survival [8] and decreases postsurgical in-hospital mortality [9].

However, the ideal time for initiation of RRT after cardiac surgery remains uncertain. Several studies have examined this topic [10–13], but the clinical complexity of this condition and the presence of multiple underlying causes have prevented the development of clear guidelines regarding the optimal time for initiation of RRT in these patients. Two meta-analyses have examined this topic [14, 15] and reported a benefit of earlier initiation of RRT for these patients, but these conclusions were based on many low-quality observational studies.

To address these knowledge gaps, we conducted a meta-analysis and used trial sequential analysis (TSA) of all relevant RCTs, to better control for type I and type II errors, to compare the outcomes of early versus late initiation of RRT in patients with CSA-AKI.

## 2. Methods

The present meta-analysis was conducted and reported according to the Preferred Reporting Items for Systematic Reviews and Meta-analyses Statement (PRISMA)** (Supplementary Materials **(available here)**) **[16]. The review protocol was registered at the PROSPERO registry of systematic reviews on February 2018 (registry number CRD42018084465).

### 2.1. Data Sources

We systematically searched PubMed, the Cochrane Library, ISI Web of Knowledge, and Embase from inception to June 2018. A basic search was performed using the following keywords: (“renal replacement therapy” OR “renal replacement” OR “hemodialysis” OR “hemofiltration” OR “dialysis” OR “dialyzed”, OR “dialyzing”) AND (“acute kidney injury” OR “AKI” OR “acute renal failure” OR “ARF” OR “acute kidney disease” OR “acute kidney stress”) AND (“cardiac surgery” OR “heart surgery” OR “coronary artery bypass grafting” OR “heart valve” OR “cardiopulmonary bypass”) AND (“early” OR “late” OR “time”). There were no language restrictions. Additional studies were identified by reviewing the reference lists of relevant articles.

### 2.2. Eligibility Criteria

Two reviewers (GLL and ZC) independently evaluated the studies for their eligibility. In cases of disagreement, a consensus was reached by discussion or consultation with a third reviewer (JC). All eligible studies were randomized clinical trials that examined early RRT initiation in adult patients (*⩾*18 years-old) with CSA-AKI; had AKI mortality data; and provided clear comparisons of early versus late initiation of RRT on mortality or other clinically relevant secondary outcomes. The following types of publications were excluded: reviews, correspondences, editorials, meeting abstracts, expert opinions, animal experiments, studies that provided insufficient information for extraction of data, studies on noncardiac surgery, non-RCTs, and pediatric studies.

### 2.3. Data Extraction

Two reviewers (GLL and DT) independently extracted the study characteristics and data from each eligible study, including the first author's name, year of publication, country of origin, study period, RRT modality, mean serum creatinine (SCr) level, number of patients, percentage of males, mean age, follow-up period, and definition of early and late RRT. Whenever possible, outcome data were extracted for the subgroups of patients with CSA-AKI, separately. Mean creatinine level and publication year were recoded as “low” or “high” based on whether they fell below or above the median value of the entire set of studies. The primary outcome was all-cause mortality, including in-hospital mortality, 14-day mortality, and 30-day mortality. The secondary outcomes were length of stay (LOS) in an intensive care unit (ICU) and LOS in a hospital.

### 2.4. Quality Assessment

The Cochrane Risk of Bias Tool was used to assess the quality of individual studies, in accordance with the* Cochrane Handbook for Systematic Reviews of Interventions* [17]. This tool considers selection bias, performance bias, attrition bias, detection bias, reporting bias, and other potential sources of bias. The overall risk of bias for each study was evaluated and rated as “low” when the risk of bias was low in all key domains; “unclear” when the risk of bias was low or unclear in all key domains; and “high” when the risk of bias was high in one or more key domains [18]. These assessments were performed independently by two reviewers (GLL and ZC), and disagreements were resolved by consensus (JC).

### 2.5. Statistical Analyses

RevMan 5.3 software from the Cochrane Collaboration was used for the meta-analysis. Relative risk (RR) was used to estimate the association between initiation time of RRT with mortality in patients with CSA-AKI. Mean differences (MDs) with 95% CIs for continuous outcomes were summarized to evaluate the associations between the time of initiation of RRT and LOS in the ICU and LOS in the hospital. When *P* < 0.05 or* I*^2^ > 50%, substantial heterogeneity was considered to exist, and the random-effects model was applied to estimate the summary RR, MDs, and 95% CI; otherwise a fixed effects model was applied [19].

Study heterogeneity in a meta-analysis, indicating the variability of the included studies, was determined using the Q test, *P* value, and* I*^2^ index, and classified as low (*I*^2^ < 50%), moderate (50% <* I*^2^ <75%), or high (*I*^2^ < 75%) [20].

Sensitivity and subgroup analyses were also conducted to determine the sources of variability among numerous factors. In addition, single covariate random-effects meta-regression was used to identify sources of variation, and a funnel plot was used to screen for potential publication bias.

### 2.6. Grading the Quality of Evidence

Two investigators independently assessed the quality of evidence for the primary outcome using the Grading of Recommendations Assessment, Development, and Evaluation (GRADE) methodology, with GRADE Pro-version 3.6 software. The quality of evidence was classified as high, moderate, low, or very low based on assessments of the risk of bias, inconsistency, indirectness, imprecision, and publication bias.

### 2.7. Trial Sequential Analysis

TSA was used to evaluate the cumulative effect of randomized trials on mortality. In this procedure, Z-curves were constructed for the primary outcome, and an alpha value at a conventional threshold was used to determine significance. Adjusted significance trial sequential monitoring boundaries were constructed using the O'Brien-Fleming alpha spending method, with the assumption that significance testing may have been performed each time a new trial is sequentially added to the meta-analysis [21]. The required information size was estimated using a two-sided *α* of 0.05 and a *β* of 0.20 (power: 80%). A risk ratio reduction of 20% was selected, because this is compatible with many trials of CSA-AKI, and it represents an absolute mortality difference of about 10% to 15%, a reasonable effect size. The open-source TSA software (version 0.9.5.10 Beta) was used for these analyses [22].

## 3. Results

### 3.1. Identification of Studies

**Figure 1:**
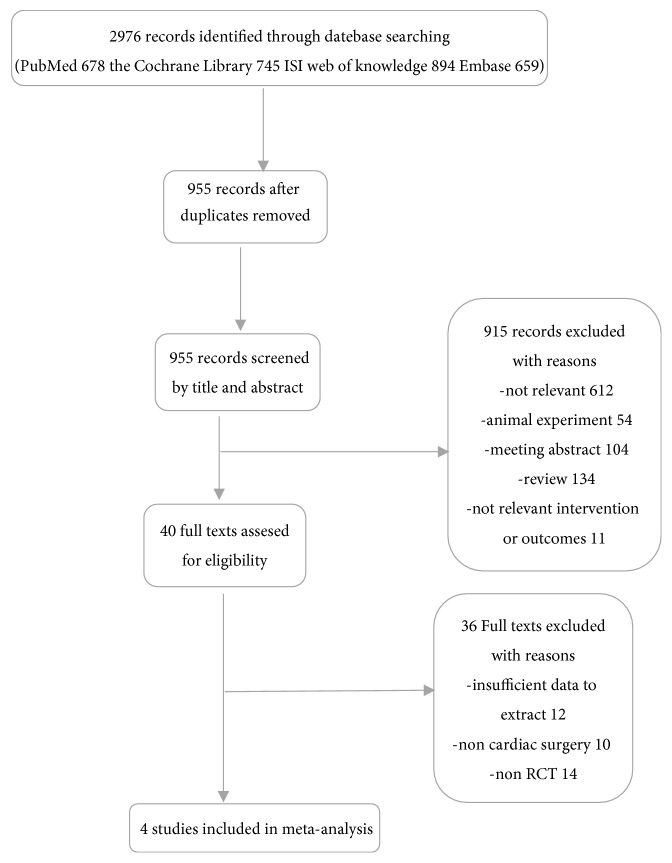
The flaw chart of included studies in the meta-analysis.

Our initial search identified 678 records from PubMed, 745 from the Cochrane Library, 894 from the ISI Web of Knowledge, and 659 from Embase (Figure 1). After removal of 2021 duplicates, we examined the titles and abstracts of the remaining 955 papers and eliminated 915 of them based on our eligibility criteria. Then, we thoroughly examined 40 articles by reading the full texts. We ultimately included 4 RCTs in the meta-analysis [10–13].

### 3.2. Characteristics of the Included Studies

The 4 included RCTs examined a total of 355 patients and were published from 2003 to 2015 (Table 1). The studies were conducted in Turkey [10], Japan [11], Italy [12], and France [13]. The RRT modality varied significantly among the four studies; three studies used continuous vena-venous hemofiltration (CVVH) [11–13] and one study used intermittent hemodialysis (IHD) [10]. The sample sizes ranged from 28 to 224, and the mean patient age ranged from 54 to 68 years. Three studies had more male than female patients [10, 11, 13]. All studies reported follow-up time, including in-hospital mortality, 14-day mortality, and 30-day mortality.

The different studies used different criteria to define early and late RRT (Table 2), so we adopted broad definitions of early and late RRT by use of different cutoffs (e.g., within a defined time after cardiac surgery, or development of urine output, or a biochemical “start time” such as SCr, etc.). Thus, we classified the 4 articles into three groups according to early RRT initiation within 12 h, within 24 h and within 48 h.

Table 3 summarizes the secondary outcomes (means and standard deviations of LOS in the ICU and LOS in the hospital).

### 3.3. Results of the Quality Assessment

Table 4 shows the quality assessment of the included studies. One study had a high risk of bias because of termination ahead of schedule [10]. Two studies had a moderate risk of bias because they did not perform “blinding of participants and personnel” [11, 12]. The remaining study had a low risk for bias [13].

### 3.4. Primary Outcomes

**Figure 2:**
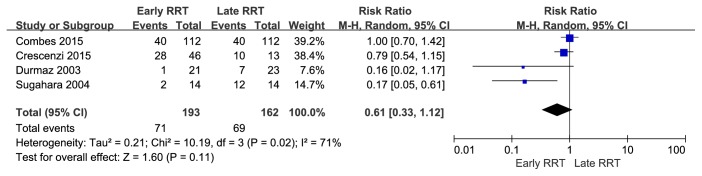
Forest plots of all 4 studies showed evidence of survival advantage of early renal replacement therapy initiation compared to late in analysis of mortality in patients with CSA-AKI.

**Figure 3:**
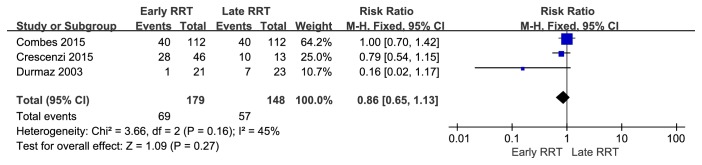
Sensitivity analysis by excluding study by Sugahara et al.

The pooled mortality rates were 36.8% (71 of 193) for patients receiving early RRT and 42.6% (69 of 162) for patients receiving late RRT. Pooled estimates indicated no significant survival benefit for early RRT (RR = 0.61, 95% CI = 0.33 to 1.12) (Figure 2). Given the moderate heterogeneity (*I*^2^ = 71%) of the four studies, we also performed a sensitivity analysis. Thus, omission of one study [11] led to an increased pooled RR (0.86, 95% CI = 0.65 to 1.13) and a decreased heterogeneity (*I*^2^ = 45%) (Figure 3). The results of other sensitivity on the basis of different standards are shown in** Supplementary Table**.

**Figure 4:**
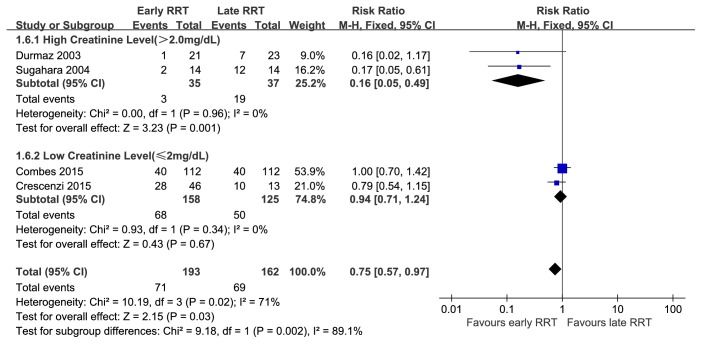
Subgroup analysis-mean creatinine level, evaluating survival benefit of early renal replacement therapy initiation compared to late in analysis of mortality in patients with CSA-AKI.

We also performed subgroup analyses based on mean SCr level to account for study heterogeneity. These results indicate that patients with a high SCr level (>2 mg/dL) who received early RRT had a pooled RR of 0.16 (95% CI = 0.05 to 0.46;* I*^2^ = 0) and those with a low SCr level (⩽2 mg/dL) who received early RRT had a pooled RR of 0.90 (95% CI = 0.69 to 1.16;* I*^2^ = 0) (Figure 4). Additionally, another subgroup analysis was conducted based on publication year, which also could explain the heterogeneity. In old studies (before 2010), the pooled RR was 0.16 (95% CI=0.05–0.46; *I*^2^ = 0). In recent studies (from 2010), the pooled RR was 0.94(95% CI=0.71–1.24; *I*^2^ = 0) (**Supplementary Table**).

**Figure 5:**
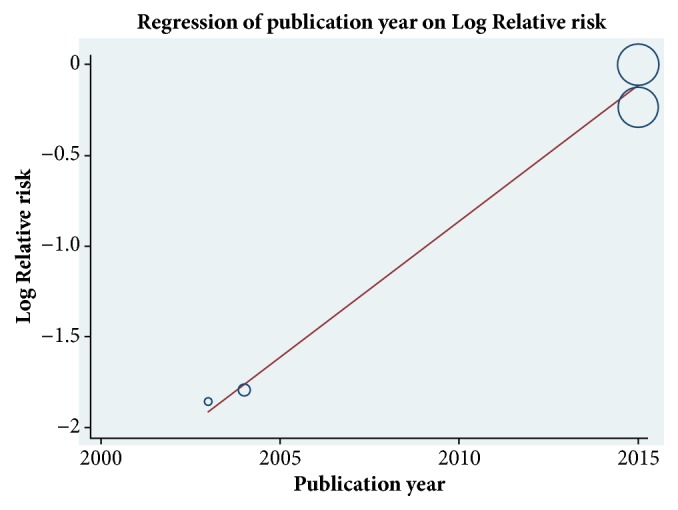
Random-effects meta-regression analysis showing the relationship between the relative risk and publication year. The size of the circles is inversely proportional to the size of the result study variance, so that more precise studies have larger circles.

**Figure 6:**
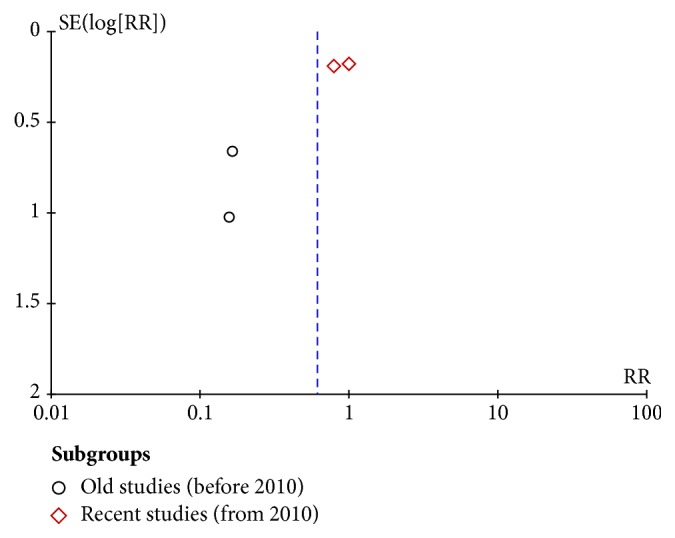
Assessment of publication bias using a funnel plot.

To identify other possible reasons for the heterogeneity, we performed a meta-regression analysis. The results publication year may be a main source of study heterogeneity (*P* = 0.037) (Figure 5). In contrast, the study period, study design (single-center or multicenter), RRT modality (IHD or CVVH), number of patients, percentage of males, mean patient age, and follow-up duration appear unrelated to study heterogeneity. A funnel plot indicated no evidence of a significant publication bias (Figure 6).

**Figure 7:**
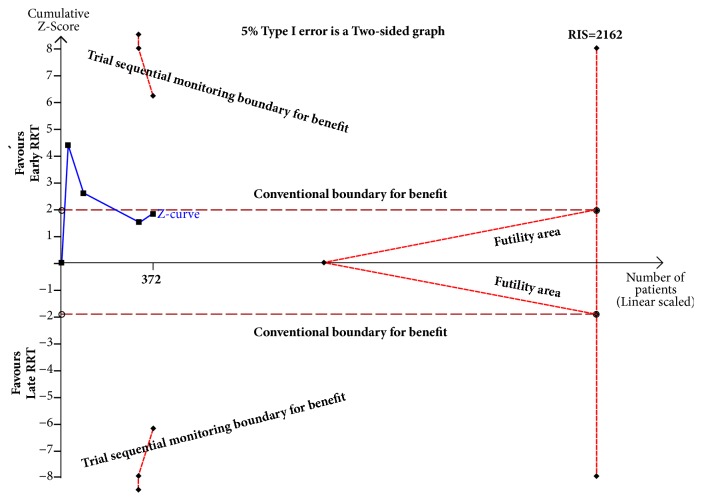
Trial sequential analysis for mortality in randomized controlled trials: a relative risk of 0.61, two-sided boundary, incidence of 42.6% in late RRT, incidence of 36.8% in early RRT, a low bias estimated relative risk reduction of 80%, *α* of 5%, and power of 80% were set. There is an estimated required information size (RIS) of 2162 randomized patients that are not reached. The boundaries for benefit are not crossed and no effect on mortality is observed; the Z-curve is parallel to the boundary of the early RRT.

Our TSA analysis indicated the cumulative Z-curve did not cross the conventional boundary for benefit and did not enter the futility boundary. A TSA sensitivity analysis that included all trials indicated that the diversity-adjusted required information size was 2162 (Figure 7).  Table 5 shows the GRADE evidence profiles for primary outcomes. Based on GRADE, the quality of the evidence was very low (Table 5).

### 3.5. Secondary Outcomes

**Figure 8:**
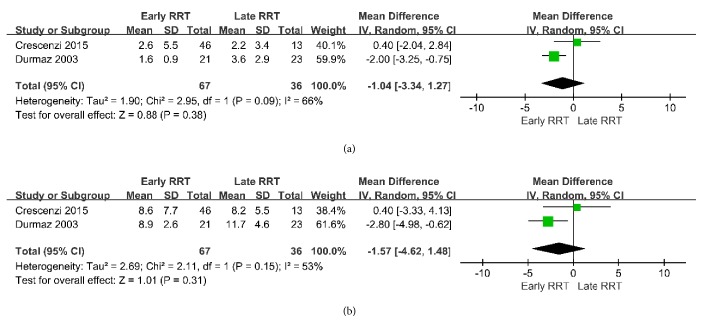
Forest plot for ICU Length of stay and hospital Length of stay.

The mean weighted LOS in the ICU was 2.0 days (n = 67) in the early RRT group and 3.0 days (n = 36) in the late RRT group; the mean weighted LOS in the hospital was 8.8 days (n = 67) in the early RRT group and 10.4 days (n = 36) in the late RRT group. Pooled analysis indicated the two groups had no significant differences in these parameters (ICU: MD = −1.04 day, 95% CI = −3.34 to 1.27; hospital: MD = −1.57 day, 95% CI = −4.62 to 1.48 (Figure 8).

## 4. Discussion

Our meta-analysis examined 4 RCTs that enrolled 335 patients who received RRT for CSA-AKI. There were 71 of 193 deaths (36.8%) in the early RRT group and 69 of 162 deaths (42.6%) in the late RRT group. Our statistical analysis showed that early initiation of RRT had no impact on survival, LOS in the ICU, or LOS in the hospital.

CSA-AKI is the most common clinically important complication in adult patients undergoing open-heart surgery and has a strong impact on short- and long-term morbidity and mortality [23, 24]. Previous research indicated that 2.1% of patients receiving open-heart surgery will develop severe AKI requiring RRT [25], and the mortality rate of these patients ranges from 36 to 78% [26]. Although RRT considerably increases the complexity of treatment for these patients, the best time for initiation of RRT remains a subject of debate [27, 28].

Early initiation of RRT may provide better control of fluid and electrolyte balance, superior acid-base homeostasis, increased removal of uremic waste, and prevention of subsequent complications attributable to AKI [29]. Furthermore, early RRT could potentially limit the kidney-specific and remote organ injuries that result from fluid overload, electrolyte imbalance, and systemic inflammation [30]. However, early RRT may also increase the risk of hemodynamic instability, anticoagulation-induced bleeding, blood-stream infection, and inflammatory or oxidative stress due to the bioincompatibility of the dialyzer membranes. Late initiation of RRT may allow more time for hemodynamic optimization prior to RRT and may prevent the need for RRT and its associated complications [31].

Our meta-analysis confirmed that early initiation of RRT had no impact on the outcomes of patients with CSA-AKI. In addition, our TSA showed that the Z-curve did not cross any of the boundaries, the required information size was not achieved, the evidence needed to reach a conclusion was insufficient, and more trials were needed to confirm the results. Our pooled analysis also showed no significant benefit of early RRT on LOS in the ICU or LOS in the hospital. Nevertheless, one subgroup analysis indicated that early initiation of RRT based on SCr level decreased mortality. This confirms another study which found that SCr level should be used as a key variable for initiation of RRT [32]. A possible explanation is that an elevated SCr level is the best indicator of renal failure that is severe enough to require RRT [33].

Notably, the studies were conducted over a wide range of time, during which the management of AKI patients has changed a lot. In the past decade the KDIGO Clinical Practice Guideline contributed to standardize and improve AKI treatment [34]. More recent studies published after 2010 failed to show a significant survival benefit from early RRT treatment, while a reduction in mortality was shown by older studies. Allegedly, the effect of early RRT therapy was blunted when administered as a part of a more comprehensive and standardized intervention.

Several previous systematic reviews have also compared early* versus *late RRT in different clinical settings (cardiac surgery and ICU) and by inclusion of studies with different designs (cohort studies and RCTs) [14, 15, 35–37]. Our analysis is unique because we only examined RCTs that examined adults in cardiac surgery. Second, we registered the protocol of our study on PROSPERO to increase the transparency and assure the quality of the meta-analysis. Third, we used TSA to confirm the conclusions and to estimate the possible effect using a more conservative approach. Finally, we analyzed the level of evidence using the GRADE approach, which classifies studies as having high, moderate, low, or very low quality of evidence.

We examined the sources of study heterogeneity by performing a sensitivity analysis with serial exclusion of individual studies. After omitting one study [11], it only could account for the segmental heterogeneity. In addition, we performed subgroup analyses based on mean SCr level. The present subgroup investigated could account for all heterogeneity. We also examined other possible reasons for study heterogeneity by performing metaregression analysis. Our results indicate the main source of heterogeneity may be publication year. Analysis of the high heterogeneity of LOS in the ICU and hospital indicated that may have been because not all articles provided original data (means and standard deviations).

Although many studies have investigated the optimal timing for initiation of RRT in patients with CSA-AKI, the results remain controversial. Initiation of RRT, to some extent, depends on SCr level and urine output, namely, the Kidney Disease: Improving Global Outcomes (KDIGO) criteria [34]. Previously reported preoperative risk factors for postoperative acute renal dysfunction requiring transient or permanent RRT were age [38], female sex [38], preoperative heart failure [38], diabetes [39], hypertension [40], anemia [41], cardiopulmonary bypass (CPB) [42], and preexisting renal dysfunction [40], especially in patients undergoing emergency surgery while clinically unstable [41]. AKI is not a specific syndrome, but is a complex pathophysiological process, especially following cardiac surgery [43], so it cannot be described by a single clinical measure. Therefore, the time for initiation of RRT must consider other clinical information. In the future, decisions regarding the optimal timing for initiation of RRT in patients with CSA-AKI should consider all the clinical data of patients.

There were several limitations of this study. First, we identified no information regarding the association of other factors with mortality, so cannot comment on differences in outcomes from a single intervention (early* versus* late initiation of RRT). Second, the definition of “early RRT” varied among the included studies, and this may have led to differences in the requirements for RRT and their therapeutic impact. Third, the sample sizes of each of the 4 included RCTs were relatively small. All outcome data, including the effect of early* versus* late initiation of RRT on mortality and the secondary outcomes, only considered 2 RCTs, insufficient to guide clinical practice. It is necessary to perform large, multicentered RCTs to confirm the results of this meta-analysis.

## 5. Conclusion

The time of initiation of RRT after cardiac surgery in patients with CSA-AKI had no apparent effect on mortality, LOS in the ICU, or LOS in the hospital. Due to limited study and not enough patient sample, large-scale, multicenter, prospective RCTs are needed to confirm the benefit of reduced mortality associated with early initiation of RRT.

## Figures and Tables

**Table 1:** Characteristics of the studies included in the meta-analysis.

**Reference**	**Nation**	**Study ** **period**	**Study ** **design**	**RRT ** **modality**	**Mean Creatinine Level(mg/dL)**		**Patients ** **(N.)**	**Male ** **Early/Late** **(**%**)**	**Mean age Early/Late (years)**	**Follow up ** **(days)**
					**Early RRT**	**Late RRT**				
**Durmaz 2003 [10]**	Turkey	1999-2001	Single- center	IHD	3.1(1.0)	4.3(1.1)	44	76/83	58/54	In- hospital mortality
**Sugahara 2004 [11]**	Japan	1995-1997	Single- center	CVVH	2.9(0.3)	3.0(0.2)	28	64/64	65/64	14-day mortality
**Crescenzi 2015 [12]**	Italy	2011-2013	Single- center	CVVH	1.7(0.9)	1.8(0.9)	59	28/31	68/ 68	30-day mortality
**Combes 2015 [13]**	France	2009-2012	Multicenter	CVVH	1.1(0.3)	1.1(0.3)	224	79/80	61/58	30-day mortality

IHD=intermittent hemodialysis, CVVH=continuous vena-venous hemofiltration, and RRT= renal replacement therapy.

**Table 2:** Definition of early and late RRT in studies included in the meta-analysis.

**Reference ** **(year)**	**KDIGO ** **criteria**	**Early RRT**	**Late RRT**
**Durmaz 2003 [10]**	KDIGO 1	Serum Cr rise >10% from pre-op level within 48 h of surgery	Serum Cr rise >50% from pre-op level or UOP <400 mL over 24 h of surgery

**Sugahara 2004 [11]**	KDIGO 2	Within 12 h of UOP <30 mL/h or urine output <750 ml/day	After 12 h of UOP <20 mL/h or urine output <500 ml/day

**Crescenzi 2015 [12]**	KDIGO 1	Within 12 h of UOP <0.5 mL/kg/h	After 12 h on the basis of persistent (>6 h of UOP <0.5 mL/kg/h) oliguria

**Combes 2015 [13]**	Unclassified	RRT initiation within 24 h post cardiac surgery in shock requiring high-dose catecholamine	Classic indication for RRT, life-threatening metabolic derangements

**KDIGO= kidney disease: improving global outcomes, RRT= renal replacement therapy, Cr=creatinine, pre-op=preoperative, and UOP=urine output.**

KDIGO 1: 1.5–1.9 times baseline or *⩾* 26.5 umol/L (0.3 mg/dl) increase in creatinine within 48 or UOP <0.5 ml/kg/h for 6–12 h.

KDIGO 2:2.0–2.9 times baseline increase in creatinine or UOP <0.5 ml/kg/h for > 12 h.

KDIGO 3: 3.0 times baseline or creatinine *⩾* 354 umol/L(4.0 mg/dl) or UPO <0.3 ml/kg/h for > 24 h or anuria for *⩾* 12 h.

**Table 3:** Secondary outcomes of early versus late RRT in patients with AKI after cardiac surgery.

**Reference ** **(year)**	**ICU LOS (days)**		** Hospital LOS (days)**	
	**Early RRT**	**Late RRT**	**Early RRT**	**Late RRT**
**Durmaz 2003 [10]**	1.6 ± 0.9	3.6 ± 2.9	8.9 ± 2.6	11.7 ± 4.8
**Sugahara 2004 [11]**	NR	NR	NR	NR
**Crescenzi 2015 [12]**	2.6 ± 5.5	2.2 ± 3.4	8.6 ± 7.7	8.2 ± 5.5
**Combes 2015 [13]**	NR	NR	NR	NR

Data are reported as mean ± standard deviation or median. RRT =renal replacement therapy, AKI=acute kidney injury, ICU=intensive care unit, LOS=length of stay, and NR= not reported.

**Table 4:** Quality assessment for randomized controlled trials.

Reference	Sequence Generation	Allocation Concealment	Blinding	Incomplete outcome data addressed	Free of selective reporting	Free of other Bias	Concurrent therapies similar	Overall risk of bias
Durmaz et al. 2003 [10]	Yes	Unclear	Unclear	Unclear	Yes	Yes	Yes	High
Sugahara et al. 2004 [11]	Yes	Yes	Unclear	Unclear	Yes	Yes	Yes	Moderate
Crescenzi et al. 2015 [12]	Yes	Unclear	Unclear	Yes	Yes	Yes	Yes	Moderate
Combes et al. 2015 [13]	Yes	Yes	Yes	Yes	Yes	Yes	Yes	Low

**Table 5:** Summary of findings table.

**Early RRT versus Late RRT in patients with cardiac surgery-associated acute kidney injury**						
**Patient or population:** patients with cardiac surgery-associated acute kidney injury **Settings:** Intensive care medicine **Intervention:** Early RRT **Comparison: **Late RRT						

**Outcomes**	**Illustrative comparative risks** **∗** ** (95**%** CI)**		**Relative effect** **(95**%** CI)**	**No of Participants** **(studies)**	**Quality of the evidence** **(GRADE)**	**Comments**
	Assumed risk	Corresponding risk				
	**Late RRT**	**Early RRT**				

**Mortality** Follow-up: 14-30 days	**Study population**		**RR 0.61** (0.33 to 1.12)	355(4 studies)	⊕⊝⊝⊝**very low**	
	**426 per 1000**	**260 per 1000** (141 to 477)				
	**Moderate**					
	**426 per 1000**	**260 per 1000** (141 to 477)				

*∗*The basis for the **assumed risk** (e.g., the median control group risk across studies) is provided in footnotes. The **corresponding risk** (and its 95% confidence interval) is based on the assumed risk in the comparison group and the **relative effect** of the intervention (and its 95% CI). **RRT**=Renal replacement therapy, **CI**=Confidence interval, **RR**=Risk ratio.						

GRADE Working Group grades of evidence **High quality:** Further research is very unlikely to change our confidence in the estimate of effect. **Moderate quality:** Further research is likely to have an important impact on our confidence in the estimate of effect and may change the estimate. **Low quality:** Further research is very likely to have an important impact on our confidence in the estimate of effect and is likely to change the estimate. **Very low quality:** We are very uncertain about the estimate.						

## Data Availability

The data used to support the findings of this study are available from the corresponding author upon request.

## Abbreviations

RRT: Renal replacement therapy
CSA-AKI: Cardiac surgery-associated acute kidney injury
RCTs: Randomized clinical trials
TSA: Trial sequential analysis
GRADE: Grading of Recommendations Assessment, Development, and Evaluation
RR: Relative risk
CI: Confidence interval
ICU: Intensive care unit
LOS: Length of stay
MD: Mean difference
AKI: Acute kidney injury
CVVH: Continuous vena-venous hemofiltration
IHD: Intermittent hemodialysis
RIS: Required information size
KDIGO: Kidney Disease: Improving Global Outcomes
CPB: Cardiopulmonary bypass.

## Supplementary Materials

Supplementary Materials Supplementary Table: results of sensitivity and subgroup analysis based on different standards.
