# Supplementary material for: Impact of Early versus Late Initiation of Renal Replacement Therapy in Patients with Cardiac Surgery-Associated Acute Kidney Injury: Meta-Analysis with Trial Sequential Analysis of Randomized Controlled Trials
Source: Biomed Res Int. 2018 Dec 18;2018:6942829. doi: 10.1155/2018/6942829 (PMC6312615; doi:10.1155/2018/6942829)
Supplement: Supplementary Materials — Supplementary Table: results of sensitivity and subgroup analysis based on different standards. [file 6942829.f1.zip › Supplementary Table.docx]

**Supplementary Table. *Results of sensitivity and subgroup analysis based on different standards***

| Analysis |  | K | N | RR [95% CI] | *P* | Study heterogeneity | | | | P(between-group comparison) |
| --- | --- | --- | --- | --- | --- | --- | --- | --- | --- | --- |
|  |  |  |  |  |  | **Chi^2^** | ***df*** | **I^2^ (%)** | ***P*** |  |
| Sensitivity  Analysis | Study design |  | | | | | | | |  |
|  | Single-center | **3** | **131** | **0.31 [0.06, 1.61]** | **0.16** | **11.96** | **2** | **83** | **0.003** |  |
|  | RRT modality |  | | | | | | | |  |
|  | CVVH | **3** | **311** | **0.71 [0.40, 1.24]** | **0.23** | **7.11** | **2** | **71** | **0.03** |  |
|  | Follow up duration |  | | | | | | | |  |
|  | 30-day | **2** | **283** | **0.90 [0.69, 1.16]** | **0.41** | **0.93** | **1** | **0** | **0.34** |  |
|  | KDIGO criteria |  |  |  |  |  |  |  |  |  |
|  | KDIGO 1 | **2** | **103** | **0.43 [0.06, 2.94]** | **0.39** | **3.77** | **1** | **73** | **0.05** |  |
|  | Time of early RRT |  | | | | | | | |  |
|  | Within 12h | **2** | **87** | **0.40 [0.06, 2.56]** | **0.33** | **7.71** | **1** | **87** | **0.005** |  |
| Subgroup analysis | Publication year |  | | | | | | | | **0.003** |
|  | Old studies (before 2010) | **2** | **72** | **0.16 [0.05, 0.49]** | **0.001** | **0** | **1** | **0** | **0.96** |  |
|  | Recent studies (from 2010) | **2** | **283** | **0.90 [0.69, 1.16]** | **0.41** | **0.93** | **1** | **0** | **0.34** |  |

**Abbreviations: K=number of studies, N= number of participants, RR=** **relative risk, CI=** **confidence interval, RRT= renal replacement therapy, CVVH=continuous vena-venous hemofiltration,** **KDIGO= kidney disease: improving global outcomes;**
