# Supplementary material for: Impact of Early versus Late Initiation of Renal Replacement Therapy in Patients with Cardiac Surgery-Associated Acute Kidney Injury: Meta-Analysis with Trial Sequential Analysis of Randomized Controlled Trials
Source: Biomed Res Int. 2018 Dec 18;2018:6942829. doi: 10.1155/2018/6942829 (PMC6312615; doi:10.1155/2018/6942829)
Supplement: Supplementary Materials — Supplementary Table: results of sensitivity and subgroup analysis based on different standards. [file 6942829.f1.zip › PRISMA Checklist.doc]

| **Section/topic** | **#** | **Checklist item** | **Reported on page #** |
| --- | --- | --- | --- |
| **TITLE** | | |  |
| Title | 1 | Impact of early versus late initiation of renal replacement therapy in patients with cardiac surgery-associated acute kidney injury: meta-analysis with trial sequential analysis of randomized controlled trials | 1 |
| **ABSTRACT** | | |  |
| Structured summary | 2 | Background: Previous studies have examined the effect of the initiation time of renal replacement therapy (RRT) in patients with cardiac surgery-associated acute kidney injury (CSA-AKI), but the findings remain controversial. The aim of this meta-analysis was to systematically and quantitatively compare the impact of early versus late initiation of RRT on the outcome of patients with CSA-AKI.  Methods: Four databases (PubMed, the Cochrane Library, ISI web of knowledge, and Embase) were systematically searched from inception to June 2018 for randomized clinical trials (RCTs). Two investigators independently performed the literature search, study selection, data extraction, and quality evaluation. Meta-analysis and trial sequential analysis (TSA) were used to examine the impact of RRT initiation time on all-cause mortality (primary outcome). The Grading of Recommendations Assessment Development and Evaluation (GRADE) was used to evaluate the level of evidence.  Results: We identified 4 RCTs with 355 patients that were eligible for inclusion. Pooled analyses indicated no difference in mortality for patients receiving early and late initiation of RRT (relative risk [RR] = 0.61, 95% confidence interval [CI] = 0.33 to 1.12). However, the results were not confirmed by TSA. Similarly, early RRT did not reduce the length of stay (LOS) in the intensive care unit (ICU) (mean difference [MD] = -1.04; 95% CI = -3.34 to 1.27) or the LOS in the hospital (MD = -1.57; 95% CI = -4.62 to 1.48). Analysis using GRADE indicated the certainty of the body of evidence was very low for a benefit from early initiation of RRT.  Conclusion: Early initiation of RRT had no beneficial impacts on outcomes in patients with CSA-AKI. Future larger and more adequately powered prospective RCTs are needed to verify the results of this meta-analysis.  Trial registration: PROSPERO registration number: CRD42018084465. Registered on 11 February 2018 | 1 |
| **INTRODUCTION** | | |  |
| Rationale | 3 | Cardiac surgery-associated acute kidney injury (CSA-AKI) is the most common clinically severe complication in adult patients following cardiac surgery. In particular, acute kidney injury (AKI) occurs in 5% to 42% of patients after cardiac surgery (depending on how AKI is defined) and is associated with increased morbidity, length of hospital stay, cost of care, and mortality. Considering the poor prognosis of these patients and the significant costs for their medical care, urgent treatment is necessary. Renal replacement therapy (RRT) is the main life-saving treatment for these patients, because it prolongs survival and decreases post-surgical in-hospital mortality. | 1 |
| Objectives | 4 | However, the ideal time for initiation of RRT after cardiac surgery remains uncertain. Several studies have examined this topic, but the clinical complexity of this condition and the presence of multiple underlying causes have prevented the development of clear guidelines regarding the optimal time for initiation of RRT in these patients. Two meta-analyses have examined this topic and reported a benefit of earlier initiation of RRT for these patients, but these conclusions were based on many low-quality observational studies.  To address these knowledge gaps, we conducted a meta-analysis and used trial sequential analysis (TSA) of all relevant RCTs, to better control for type I and type II errors, to compare the outcomes of early versus late initiation of RRT in patients with CSA-AKI. | 2-3 |
| **METHODS** | | |  |
| Protocol and registration | 5 | The present meta-analysis was conducted and reported according to the Preferred Reporting Items for Systematic Reviews and Meta-analyses Statement (PRISMA) . The review protocol was registered at the PROSPERO registry of systematic reviews on February 2018 (registry number: CRD42018084465). | 2 |
| Eligibility criteria | 6 | All eligible studies were randomized clinical trials that examined early RRT initiation in adult patients (≥18 years-old) with CSA-AKI; had AKI mortality data; and provided clear comparisons of early versus late initiation of RRT on mortality or other clinically relevant secondary outcomes. | 2 |
| Information sources | 7 | PubMed, the Cochrane Library, ISI Web of Knowledge, and Embase from inception to June 2018. | 2 |
| Search | 8 | We systematically searched PubMed, the Cochrane Library, ISI Web of Knowledge, and Embase from inception to June 2018. A basic search was performed using the following keywords: (“renal replacement therapy” OR “renal replacement” OR “hemodialysis” OR “hemofiltration” OR “dialysis” OR “dialyzed”, OR “dialyzing”) AND (“acute kidney injury” OR “AKI” OR “acute renal failure” OR “ARF” OR “acute kidney disease” OR “acute kidney stress”) AND (“cardiac surgery” OR “heart surgery” OR “coronary artery bypass grafting” OR “heart valve” OR “cardiopulmonary bypass”) AND (“early” OR “late” OR “time”). There were no language restrictions. | 2 |
| Study selection | 9 | Two authors independently screened title and /or abstract produced by the search. Full-text manuscripts that were identified as relevant were obtained and then assessed independently against inclusion and exclusion criteria. Discrepancies were settled by discussion and/or by introducing other reviewers. | 2 |
| Data collection process | 10 | Two reviewers independently extracted the study characteristics and data from each eligible study, including the first author’s name, year of publication, country of origin, study period, RRT modality, mean serum creatinine (SCr) level, number of patients, percentage of males, mean age, follow-up period and definition of early and late RRT. Whenever possible, outcome data were extracted for the subgroups of patients with CSA-AKI, separately. Mean creatinine level and publication year were recoded as “low” or “high” based on whether they fell below or above the median value of the entire set of studies. The primary outcome was all-cause mortality, including in-hospital mortality, 14-day mortality, and 30-day mortality. The secondary outcomes were length of stay (LOS) in an intensive care unit (ICU) and LOS in a hospital. | 2 |
| Data items | 11 | first author’s name, year of publication, country of origin, study period, RRT modality, mean serum creatinine (SCr) level, number of patients, percentage of males, mean age, follow-up period and definition of early and late RRT. | 2 |
| Risk of bias in individual studies | 12 | The Cochrane Risk of Bias Tool was used to assess the quality of individual studies, in accordance with the Cochrane Handbook for Systematic Reviews of Interventions. This tool considers selection bias, performance bias, attrition bias, detection bias, reporting bias, and other potential sources of bias. The overall risk of bias for each study was evaluated and rated as “low” when the risk of bias was low in all key domains; “unclear” when the risk of bias was low or unclear in all key domains; and “high” when the risk of bias was high in one or more key domains. These assessments were performed independently by two reviewers, and disagreements were resolved by consensus. | 2 |
| Summary measures | 13 | RevMan 5.3 software from the Cochrane Collaboration was used for the meta-analysis. Relative risk (RR) was used to estimate the association between initiation time of RRT with mortality in patients with CSA-AKI. RR and the associated 95% confidence interval (CI) were pooled using fixed-effect (Mantel-Haenszel method) or random-effect (DerSimonian and Laird method) models. Mean differences (MDs) with 95% CIs for continuous outcomes were summarized to evaluate the associations between the time of initiation of RRT and LOS in the ICU and LOS in the hospital. | 2 |
| Synthesis of results | 14 | Study heterogeneity in a meta-analysis, indicating the variability of the included studies, was determined using the Q test, ***P*** value, and ***I***^2^ index, and classified as low (***I***^2^ < 50%), moderate (50% < ***I***^2^ <75%), or high (***I***^2^ < 75%). and a funnel plot was used to screen for potential publication bias. | 2 |

Page 1 of 2

| Section/topic | # | Checklist item | Reported on page # |
| --- | --- | --- | --- |
| Risk of bias across studies | 15 | Funnel plots were used to screen for potential publication bias | 2 |
| Additional analyses | 16 | In addition, to investigate the potential sources of heterogeneity in the eligible studies, Sensitivity and subgroup analyses were also conducted to determine the sources of variability among numerous factors. In addition, single covariate random-effects meta-regression was used to identify sources of variation. Two investigators independently assessed the quality of evidence for the primary outcome using the Grading of Recommendations Assessment, Development, and Evaluation (GRADE) methodology, with GRADE Pro-version 3.6 software. The quality of evidence was classified as high, moderate, low, or very low based on assessments of the risk of bias, inconsistency, indirectness, imprecision, and publication bias. TSA was used to evaluate the cumulative effect of randomized trials on mortality. In this procedure, Z-curves were constructed for the primary outcome, and an alpha value at a conventional threshold was used to determine significance. Adjusted significance trial sequential monitoring boundaries were constructed using the O’Brien-Fleming alpha spending method, with the assumption that significance testing may have been performed each time a new trial is sequentially added to the meta-analysis. The required information size was estimated using a two-sided α of 0.05 and a β of 0.20 (power: 80%). A risk ratio reduction of 20% was selected, because this is compatible with many trials of CSA-AKI, and it represents an absolute mortality difference of about 10% to 15%, a reasonable effect size. The open-source TSA software (version 0.9.5.10 Beta) was used for these analyses | 2-4 |
| RESULTS | | |  |
| Study selection | 17 | Our initial search identified 678 records from PubMed, 745 from the Cochrane Library, 894 from the ISI Web of Knowledge, and 659 from Embase. After removal of 2021 duplicates, we examined the titles and abstracts of the remaining 955 papers, and eliminated 915 of them based on our eligibility criteria. Then, we thoroughly examined 40 articles by reading the full texts. We ultimately included 4 RCTs in the meta-analysis. | 4 |
| Study characteristics | 18 | The 4 included RCTs examined a total of 355 patients and were published from 2003 to 2015. The studies were conducted in Turkey, Japan, Italy, and France. The RRT modality varied significantly among the four studies; three studies used continuous vena-venous hemofiltration (CVVH) and one study used intermittent hemodialysis (IHD) . The sample sizes ranged from 28 to 224, and the mean patient age ranged from 54 to 68 years. Three studies had more male than female patients. All studies reported follow-up time, including in-hospital mortality, 14-day mortality, and 30-day mortality.The different studies used different criteria to define early and late RRT, so we adopted broad definitions of early and late RRT by use of different cutoffs (e.g. within a defined time after cardiac surgery, or development of urine output, or a biochemical “start time” such as SCr, etc.). Thus, we classified the 4 articles into three groups according to early RRT initiation within 12 h, within 24 h and within 48 h.Table 3 summarizes the secondary outcomes (means and standard deviations of LOS in the ICU and LOS in the hospital). | 4-5 |
| Risk of bias within studies | 19 | Table 4 shows the quality assessment of the included studies. One study had a high risk of bias because of termination ahead of schedule. Two studies had a moderate risk of bias because they did not perform “blinding of participants and personnel”. The remaining study had a low risk for bias. | 5 |
| Results of individual studies | 20 | The pooled mortality rates were 36.8% (71 of 193) for patients receiving early RRT and 42.6% (69 of 162) for patients receiving late RRT. Pooled estimates indicated no significant survival benefit for early RRT (RR = 0.61, 95% CI = 0.33 to 1.12). | 5 |
| Synthesis of results | 21 | The pooled mortality rates were 36.8% (71 of 193) for patients receiving early RRT and 42.6% (69 of 162) for patients receiving late RRT. Pooled estimates indicated no significant survival benefit for early RRT (RR = 0.61, 95% CI = 0.33 to 1.12). | 5 |
| Risk of bias across studies | 22 | Publication bias was considered to be no obvious | 5 |
| Additional analysis | 23 | Given the moderate heterogeneity (***I***^2^ = 71%) of the four studies, we also performed a sensitivity analysis. Thus, omission of one study led to an increased pooled RR (0.83, 95% CI = 0.56 to 1.25) and a decreased heterogeneity (I^2^ = 45%). We also performed subgroup analyses based on mean SCr level to account for study heterogeneity. These results indicate that patients with a high SCr level (>2 mg/dL) who received early RRT had a pooled RR of 0.16 (95% CI = 0.05 to 0.46; ***I***^2^ = 0) and those with a low SCr level (≤2 mg/dL) who received early RRT had a pooled RR of 0.90 (95% CI = 0.69 to 1.16; ***I***^2^ = 0). Additionally, another subgroup analysis were conducted based on publication year, which also could explain the heterogeneity. In old studies (before 2010), the pooled RR was 0.16(95% CI=0.05–0.46; ***I***^2^ =0).In recent studies (from 2010), the pooled RR was 0.90(95% CI=0.69–1.16; ***I***^2^ =0). To identify other possible reasons for the heterogeneity, we performed a meta-regression analysis. The results publication year may be a main source of study heterogeneity (***P*** = 0.037). In contrast, the study period, study design (single-center or multi-center), RRT modality (IHD or CVVH), number of patients, percentage of males, mean patient age, and follow up duration appear unrelated to study heterogeneity. A funnel plot indicated no evidence of a significant publication bias. Our TSA analysis indicated the cumulative Z-curve did not cross the conventional boundary for benefit and did not enter the futility boundary. A TSA sensitivity analysis that included all trials indicated that the diversity-adjusted required information size was 2162. Table 5 shows the GRADE evidence profiles for primary outcomes. Based on GRADE, the quality of the evidence was very low. | 5-6 |
| **DISCUSSION** | | |  |
| Summary of evidence | 24 | Our meta-analysis examined 4 RCTs that enrolled 335 patients who received RRT for CSA-AKI. There were 71 of 193 deaths (36.8%) in the early RRT group, and 69 of 162 deaths (42.6%) in the late RRT group. Our statistical analysis showed that early initiation of RRT had no impact on survival, LOS in the ICU, or LOS in the hospital. | 6 |
| Limitations | 25 | There were several limitations of this study. First, we identified no information regarding the association of other factors with mortality, so cannot comment on differences in outcomes from a single intervention (early vs. late initiation of RRT). Second, the definition of “early RRT” varied among the included studies, and this may have led to differences in the requirements for RRT and their therapeutic impact. Third, the sample sizes of each of the 4 included RCTs were relatively small. All outcome data, including the effect of early vs. late initiation of RRT on mortality and the secondary outcomes, only considered 2 RCTs, insufficient to guide clinical practice. It is necessary to perform large, multi-centered RCTs to confirm the results of this meta-analysis. | 11 |
| Conclusions | 26 | The time of initiation of RRT after cardiac surgery in patients with CSA-AKI had no apparent effect on mortality, LOS in the ICU, or LOS in the hospital. Large-scale, multicenter, prospective RCTs are needed to confirm the results of this meta-analysis. | 11 |
| **FUNDING** | | |  |
| Funding | 27 | Not applicable. | 11 |
